# The developing hypopharyngeal microbiota in early life

**DOI:** 10.1186/s40168-016-0215-9

**Published:** 2016-12-30

**Authors:** Martin Steen Mortensen, Asker Daniel Brejnrod, Michael Roggenbuck, Waleed Abu Al-Soud, Christina Balle, Karen Angeliki Krogfelt, Jakob Stokholm, Jonathan Thorsen, Johannes Waage, Morten Arendt Rasmussen, Hans Bisgaard, Søren Johannes Sørensen

**Affiliations:** 1 Department of Biology, Section of Microbiology, University of Copenhagen, Universitetsparken 15, bldg. 1, DK2100 Copenhagen, Denmark; 2 Copenhagen Prospective Studies on Asthma in Childhood, Faculty of Health Sciences, University of Copenhagen, Copenhagen University Hospital Gentofte, 2820 Gentofte, Denmark; 3 Microbiology and Infection Control, Statens Serum Institut, Artillerivej 5, 2300 Copenhagen S, Denmark; 4 Department of Food Science, Faculty of Science, University of Copenhagen, 1958 Frederiksberg C, Denmark

**Keywords:** Airway microbiome, Infant microbiome, Microbiome development

## Abstract

**Background:**

The airways of healthy humans harbor a distinct microbial community. Perturbations in the microbial community have been associated with disease, yet little is known about the formation and development of a healthy airway microbiota in early life. Our goal was to understand the establishment of the airway microbiota within the first 3 months of life.

We investigated the hypopharyngeal microbiota in the unselected COPSAC_2010_ cohort of 700 infants, using 16S rRNA gene sequencing of hypopharyngeal aspirates from 1 week, 1 month, and 3 months of age.

**Results:**

Our analysis shows that majority of the hypopharyngeal microbiota of healthy infants belong to each individual’s core microbiota and we demonstrate five distinct community pneumotypes. Four of these pneumotypes are dominated by the genera *Staphylococcus*, *Streptococcus*, *Moraxella*, and *Corynebacterium*, respectively. Furthermore, we show temporal pneumotype changes suggesting a rapid development towards maturation of the hypopharyngeal microbiota and a significant effect from older siblings. Despite an overall common trajectory towards maturation, individual infants’ microbiota are more similar to their own, than to others, over time.

**Conclusions:**

Our findings demonstrate a consolidation of the population of indigenous bacteria in healthy airways and indicate distinct trajectories in the early development of the hypopharyngeal microbiota.

**Electronic supplementary material:**

The online version of this article (doi:10.1186/s40168-016-0215-9) contains supplementary material, which is available to authorized users.

## Background

No surfaces on the human body escape colonization by microbes; this is particularly true for the large surface area of the gut.

The human gut microbiota has been extensively studied [1]. It is shown that the gut is initially, in newborn infants, colonized by facultative anaerobe skin bacteria, followed by better adapted obligate anaerobe bacteria, when a sufficiently anaerobic environment has formed [2, 3]. During the first years of life, the gut microbiota adapts to changing diet and is over time shaped into specific enterotypes, each representing a more or less well-defined microbiota composition [4]. The gut microbiota has been shown to influence maturation of the immune system and the development of various immune-mediated diseases [5–8].

The human lungs have a larger surface area than the gut [9, 10] and similarly provide intimate contact with the host immune system. However, healthy lungs have traditionally been considered sterile and only colonized by bacteria during infections. In addition, the lower airways are difficult to access, which makes sampling invasive and uncomfortable. Recent studies have however found correlations between specific compositions of the airway microbiota and diseases such as chronic obstructive pulmonary disease, cystic fibrosis and asthma [11–13]. Studies with low numbers of healthy adults have overcome the inherent difficulties in sampling the lower airways. In these studies, lung-specific bacteria have been identified in bronchoaveolar lavage samples by comparison to multiple sites along the upper and lower airways [14]. Others have shown that there is a microbiological continuity within the airways of healthy adults, where the lower airways are distinct from the upper airways, but contains many of the same bacteria [15].

Studies of the early upper airway microbiota in infants have linked the early microbiota to disease development later in life. We have previously demonstrated that indeed 22% of neonates were asymptomatically carrying *Haemophilus influenzae*, *Streptococcus pneumoniae*, and *Moraxella catarrhalis*. These children exhibited four- to fivefold increased risk of developing asthma later in childhood [16]. Teo et al. studied the infant nasopharyngeal microbiota and found that certain bacteria correlated to acute respiratory infections and the risk of allergic sensitization and chronic wheeze [17].

More knowledge on the development of the airway microbiota in early life is a fundamental necessity for understanding the interactions between the microbiota and the developing immune system, which are expected to impact later health [18].

The present study is based on the prospective Copenhagen Prospective Studies on Asthma in Childhood 2010 (COPSAC_2010_) mother-child birth cohort [19]. Here, the hypopharyngeal microbiota of healthy infants was sampled by aspirations at 1 week, 1 month, and 3 months of age. We explore the hypopharyngeal microbiota development to determine if distinct microbial community types exist during early life.

## Results

### Sample population

In this study, we included 1788 hypopharyngeal samples collected from 695 infants at 1 week, 1 month, and 3 months after birth; samples from all time-points were included from 438 of the infants. The anthropometric and seasonal information about the infants in this study, as well as data on antibiotic use and diet, is shown in Table 1. Additionally, we have included 32 DNA extraction negative controls in our analysis.

**Table 1:** Characteristics of the study population (*n* = 695 infants)

Characteristic			Summary statistic
Anthropometrics			
Boys, *n*(%)			356 (51%)
Gestational age <36 weeks, *n*(%)			20 (3%)
C-section, *n*(%)			150 (22%)
Mother asthmatic, *n*(%)			181 (26%)
Mother antibiotic in third trimester, *n*(%)			31 (4%)
Siblings, *n*(%)			392 (56%)
Season of birth			
Spring, *n*(%)			184 (26%)
Summer, *n*(%)			149 (21%)
Fall, *n*(%)			148 (21%)
Winter, *n*(%)			2 (31%)
Clinical information	1 week	1 month	3 months
Samples, *n*	544	621	623
Antibiotic used, *n*(%)	11 (2%)	23 (4%)	34 (5%)
Only breastfed, *n*(%)	477 (88%)	473 (76%)	394 (63%)

### Low biomass samples

To ensure that our analyses were not confounded by spurious results from low biomass samples, we first analyzed the alpha diversity of negative control samples that produced sequencing reads and hypopharyngeal samples (Additional file 1: Figure S1). The DNA extraction negative controls had significantly lower observed richness than all hypopharyngeal samples, with exception of the extremely low DNA group (analysis of variance (ANOVA), *P* = 0.64), and evenness were significantly higher in the DNA extraction negative controls compared to the hypopharyngeal samples (ANOVA, *P* < 10^−7^). Having confirmed that our samples differed from the controls, we created a heatmap of the 25 most abundant OTUs, with the samples clustered by their Euclidian distances (Fig. 5). The samples did not show any clustering by extracted DNA concentration; therefore, we chose to include all samples.

### Composition of hypopharyngeal microbiota over time

The most frequent phyla were Firmicutes (61% of reads), Proteobacteria (30%), Actinobacteria (6%), and Bacteroidetes (2%) (Additional file 2: Figure S2). We observed a temporal increasing alpha diversity (Shannon's dive﻿rsity index (SDI): mean 1.09, 1.26, and 1.44, ANOVA *P* < 10^−5^ and *P* < 10^−6^, respectively; Observed richness: mean 55.2, 77.1 and 80.2, ANOVA *P* < 10^−7^ and *P* = 0.17, respectively) and seasonal variation; Observed richness was high during spring (March, April, and June) and low during summer (June, July, and August) (ANOVA *P* = 0.006, 10^−5^, and 0.02, respectively), SDI tended to be low in summer and high in winter and spring but not significantly so (Additional file 1: Table S1). There was a change in the microbiota composition between time-points: The genus *Streptococcus* almost doubled between 1 week and 1 month and then maintained that abundance at 3 months (17, 31, and 29% of reads, respectively; ANOVA *P* < 10^−7^ and *P* = 0.5, for the two time spans). *Staphylococcus* genus dominated at 1 week but decreased more than 50% between each of the following time-points (49, 22, and 10%; ANOVA *P* < 10^−7^ between all time-points). The abundance of *Moraxella* genus increased between each time-point, from 9% at 1 week, to 13% at 1 month, and to 23% at 3 months (ANOVA *P* < 0.01 and *P* < 10^−7^, for the two time spans respectively).

### Core microbiota in each infant

We investigated the stability of the microbiota composition and the abundance of OTUs present in infants at all time-points (437 infants, Additional file 1: Table S2) to determine which proportion of the infants hypopharyngeal microbiota that were from continuously present OTUs, expected colonizers, and which were transient OTUs, that were not persistent members of the infants microbiota. We defined an infant’s individual core microbiota as the OTUs present in all three samples from that infant. Each infant’s core microbiota consisted of 1–17 OTUs, most commonly Streptococcus_OTU4, with the five most abundant genera dominating the ten most common OTUs (Additional file 1: Table S3). Comparing the median abundance of the core microbiota at each time-point (1 week : 90%, 1 month : 81%, and 3 months : 63%), we found a significant decrease in the median core microbiota (Wilcoxon *P* < 0.001), reflecting increased diversity as the hypopharyngeal microbiota develops. However, OTUs already present at 1 week represented 63% of the microbiota abundance at 3 months, indicating an early establishment of a permanent resident hypopharyngeal microbiota.

### Characterization of pneumotypes

**Fig. 1:**
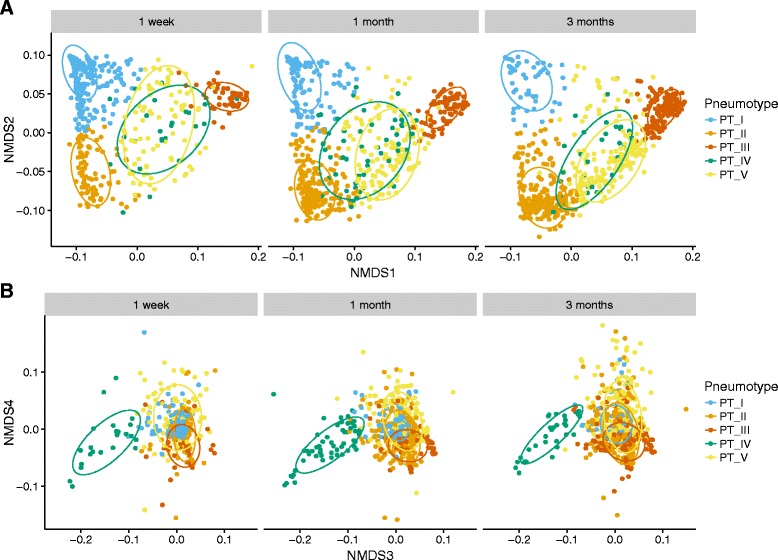
Pneumotype clusters separated in NMDS plots using 4 axes. Microbial clustering on the basis of weighted UniFrac distances, visualized by non-metric multidimensional scaling (NMDS, stress = 0.073), with ellipses encircling 75% of samples from each pneumotype. **a** Pneumotypes I, II, and III are separated on NMDS axes 1 and 2, with pneumotypes IV and V overlapping in the center of the plot. **b** On axes 3 and 4, pneumotype IV separates from the other pneumotypes. Axes’ minimum and maximum limits were fixed to exclude six outlying samples; the coordinates of these samples can be found in Additional file 1: Table S4

Having shown that bacteria with a continuous presence represent a large fraction of the hypopharyngeal microbiota we wanted to investigate how well the children cluster based on their hypopharyngeal microbiota. Distance-based clustering revealed that the samples were optimally described by five clusters, as shown in Fig. 1, henceforth referred to as pneumotype I to V, based on the average silhouette width metric. We confirmed that no other five-cluster grouping had lower within cluster distances using a permutation test (*P* < 10^−6^), thus confirming that our clustering is the optimal representation of five clusters.

**Fig. 2:**
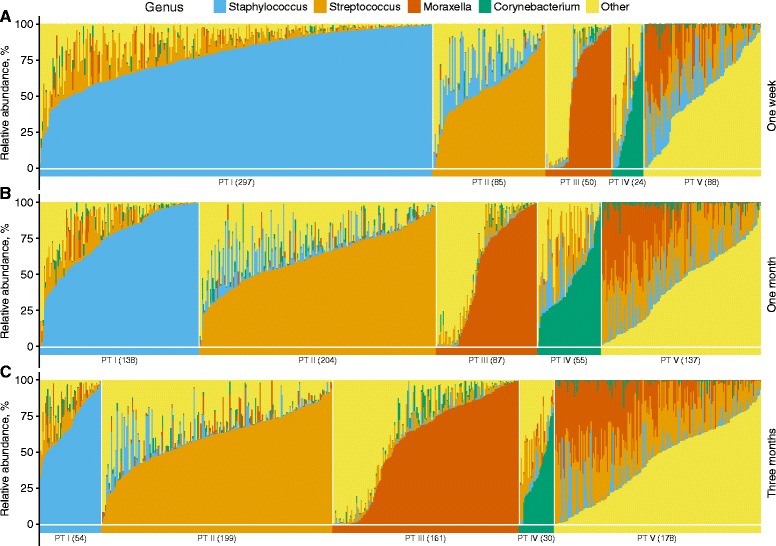
Abundance of dominant genera shows the difference between pneumotypes. Bar plot showing the abundance indicator genera in each sample, separated by time. **a** One-week samples. **b** One-month samples. **c** Three-months samples. The samples are sorted by pneumotype and within the pneumotype, by the abundance of the pneumotypes indicator genus. Pneumotype have been abbreviated as PT in this figure

Characterizing the main microbial constituents of each pneumotype using the indicator species approach [20], we found clear indicator OTUs from the genera *Staphylococcus* (pneumotype I), *Streptococcus* (pneumotype II), *Moraxella* (pneumotype III), and *Corynebacterium* (pneumotype IV) (Fig. 2; Additional file 1: Table S5). In addition to differences in main microbial constituents, pneumotypes I and III had a significantly lower SDI (ANOVA, *P* < 10^−15^), while the observed richness was significantly higher for pneumotypes II and V (ANOVA, *P* < 10^−15^).

The effects of clinical exposures on the pneumotypes were compared at each time-point separately. At any time-point, more of the infants with pneumotype III and V had siblings (65.2–80.9%), and at 3 months significantly fewer of the infants with pneumotype I had siblings (26.4%). The proportion of infants delivered by caesarean section was significantly different between the pneumotypes at 1 month, pneumotype III had a lower proportion of infants born by caesarean section (12.4%), while pneumotype IV had the highest (34.0%). The proportions of infants born by caesarean section were very different in the 1-week samples, and following a similar pattern at the 3-month time-point, these differences were not significant. The pneumotypes did not seem to have a consistent proportion of infants having received antibiotic treatment at any point before sampling. At 1 month, pneumotype IV did have a significantly higher proportion of infants that had received antibiotic treatment. Similarly, exclusively breastfeeding was not consistently correlated with the pneumotypes over time, but at 1 month a significantly lower proportion of the infants with pneumotype I or II were still exclusively breastfed. All data are shown in Table 2.

**Table 2:** Characterization of pneumotypes

	Pneumotype I	Pneumotype II	Pneumotype III	Pneumotype IV	Pneumotype V
One week, *n*	One week, *n*	87	51	24	85
Age at sampling in days, mean ± sd	8.1 ± 2.7	8.0 ± 3.7	7.6 ± 2.8	7.8 ± 3.0	8.0 ± 3.1
SDI, mean ± sd	0.83 ± 0.51	1.31 ± 0.42	0.78 ± 0.49	1.57 ± 0.63	1.74 ± 0.46
Observed Richness, mean ± sd	21.5 ± 11.4	24.3 ± 8.2	19.0 ± 8.0	23.4 ± 11.5	25.8 ± 9.6
Exclusively breastfed, *n*(%)	266 (90.8%)	72 (83.7%)	47 (92.2%)	20 (83.3%)	72 (86.7%)
Any antibiotic, *n*(%)	3 (1.0%)	2 (2.4%)	1 (2.0%)	1 (4.2%)	4 (4.9%)
Caesarean section, *n*(%)	66 (22.2%)	13 (14.9%)	8 (15.7%)	1 (4.2%)	21 (24.7%)
Siblings, *n*(%)	163 (55.1%)	47 (54.0%)	39 (76.5%)	12 (50.0%)	60 (70.6%)
Mother asthmatic, *n*(%)	76 (25.7%)	20 (23.0%)	17 (33.3%)	6 (25.0%)	25 (29.4%)
Male, *n*(%)	152 (51.2%)	47 (54.0%)	27 (52.9%)	13 (54.2%)	35 (41.2%)
One month, *n*	137	211	89	53	131
Age at sampling in days, mean ± sd	31.3 ± 4.7	32.6 ± 5.3	32.2 ± 5.8	31.1 ± 3.7	32.5 ± 5.6
SDI, mean ± sd	0.87 ± 0.55	1.41 ± 0.48	0.77 ± 0.42	1.40 ± 0.48	1.64 ± 0.43
Observed Richness, mean ± sd	22.5 ± 13.2	29.1 ± 10.8	19.1 ± 7.2	22.3 ± 8.8	30.5 ± 14.5
Exclusively breastfed, *n*(%)	96 (70.1%)	152 (72.7%)	76 (85.4%)	41 (78.8%)	108 (83.1%)
Any antibiotic, *n*(%)	3 (2.2%)	8 (3.8%)	3 (3.5%)	6 (11.8%)	3, (2.3%)
Caesarean section, *n*(%)	41 (29.9%)	47 (22.3%)	11 (12.4%)	18 (34.0%)	20 (15.3%)
Siblings, *n*(%)	68 (49.6%)	98 (46.4%)	72 (80.9%)	23 (43.4%)	99 (76.2%)
Mother asthmatic, *n*(%)	37 (27.2%)	59 (28.1%)	26 (29.5%)	14 (26.4%)	27 (20.6%)
Male, *n*(%)	69 (50.4%)	114 (54.0%)	46 (51.7%)	30 (56.6%)	61 (46.6%)
Three months Samples, *n*	54	196	165	30	178
Age at sampling in days, mean ± sd	93.0 ± 5.9	92.9 ± 6.3	92.6 ± 7.1	91.7 ± 4.6	93.9 ± 6.6
SDI, mean ± sd	1.24 ± 0.53	1.47 ± 0.50	0.97 ± 0.51	1.72 ± 0.43	1.80 ± 0.47
Observed Richness, mean ± sd	31.6 ± 10.1	34.4 ± 12.1	24.39 ± 10.5	28.7 ± 11.3	35.0 ± 13.4
Exclusively breastfed, *n*(%)	34 (64.2%)	65.5% (127)	66.2% (102)	20 (66.7%)	111 (65.7%)
Any antibiotic, *n*(%)	2 (3.8%)	13 (6.8%)	5 (3.3%)	0 (0.0%)	15 (8.9%)
Caesarean section, *n*(%)	13 (24.1%)	46 (23.5%)	28 (17.0%)	9 (30.0%)	34 (19.1%)
Siblings, *n*(%)	14 (26.4%)	85 (43.4%)	126 (76.4%)	13 (43.3%)	116 (65.2%)
Mother asthmatic, *n*(%)	14 (25.9%)	47 (24.1%)	46 (28.0%)	5 (16.7%)	51 (28.8%)
Male, *n*(%)	29 (53.7%)	99 (50.5%)	86 (52.1%)	9 (30.0%)	92 (51.7%)

### Time dependency of pneumotypes

**Fig. 3:**
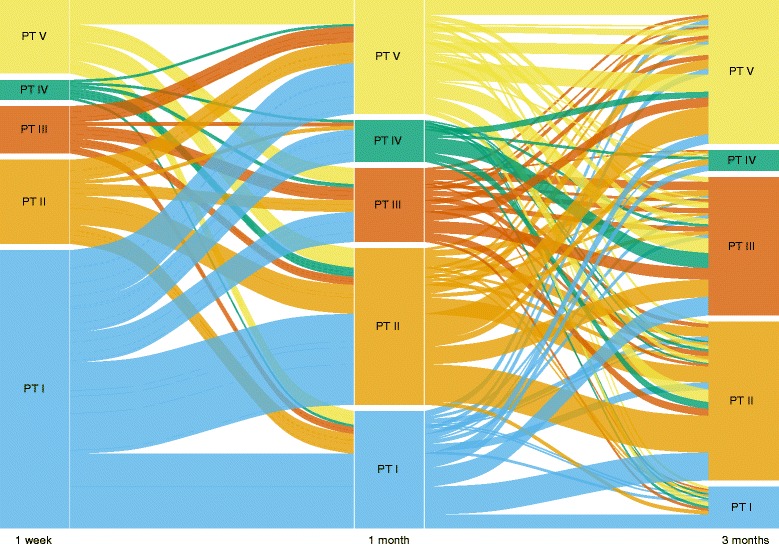
Dynamics of the infants’ pneumotype shown by an alluvial plot. Alluvial plot showing which pneumotype each infant presents over time, including the 438 infants with three samples (Additional file 1: Table S6). Each infant is represented by a line connecting their pneumotype at 1 week, through their pneumotype at 1 month, to their pneumotype at 3 months. The first part of the lines, from 1 week to 1 month, colored by their pneumotype at 1 week, and the second part of the lines, from 1 month to 3 months, colored by their pneumotype at 1 month. Pneumotype have been abbreviated as PT in this figure

There was a significant change in the distribution of samples in the five pneumotypes over time (*χ* ^2^ test, *P* < 10^−15^; Fig. 3). The number of infants with pneumotype I decreased while pneumotypes II, III, and V became more abundant. Despite these large-scale changes over time, the pneumotypes were correlated over the two time spans, 1 week vs. 1 month and 1 month vs. 3 months (*P* = 0.004 and *P* < 10^−6^, respectively). Additionally, finding the same pneumotype at 1 month and 3 months (34% of infants) was more likely than at 1 week and 1 month (28%) (*χ* ^2^ test, *P* = 0.03). To compare how the pneumotypes correlated from 1 week to 3 months, we analyzed samples from the 437 infants with samples from all time-points (Additional file 1: Table S2). Of these infants, the proportion maintaining the same pneumotype at all time-points (9.4%) was significantly higher than random (*χ* ^2^ test, *P* < 0.003). This was also true when testing each pneumotype separately, except for the less well-defined pneumotype IV (Additional file 1: Table S7).

The changes in pneumotypes over time show that the maturation of the hypopharyngeal microbiota follows a trajectory, while still being partially dependent on the initial colonization found at 1 week.

**Fig. 4:**
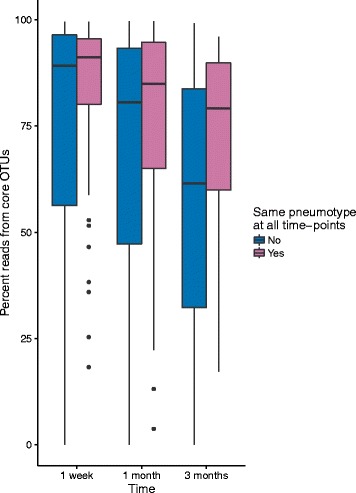
The core microbiota is more abundant in infants having one pneumotype continuously. Percentage of reads from the core microbiota over time, separated by whether or not the sample belongs to an infant, which have one pneumotype continuously. There is an increasing difference in core microbiota abundance between the two groups over time (Additional file 1: Table S8), being significantly different at 3 months (Wilcoxon rank sum test, *P* = 0.001)

The infants with stable pneumotypes had significantly higher core microbiota abundance than infants with changing pneumotypes (ANOVA, *P* = 0.006). This difference in abundance of core microbiota between the two groups increased over time (Fig. 4, Additional file 1: Table S8).

### Comparison of distance within and between infants

To confirm that this maturation of the microbiota was not solely a time-dependent change, we analyzed the ratio of the weighted UniFrac distance from each infant’s 1-week sample to 1-month sample and the distance to other infants’ 1-month samples and similarly from 1 month to 3 months. Over the two time spans, the median ratios were 0.93 and 0.87, respectively. These were confirmed to be significantly different from 1.0 by permutations of which column represented the infants’ own samples in the distance matrix (both *P* < 0.0001). When comparing 1-week samples to 3-month samples, the median ratio was 0.98 (*P* = 0.07), meaning that the distances to the infants’ own samples were not significantly lower than the distance to other infants’ samples.

Based on these distances, the development of each infant’s 1-week microbiota could not be linked directly to the microbiota at 3 months; but when including the 1-month microbiota, there was a traceable development from 1-week microbiota to 3-month microbiota.

## Discussion

We here present the first extensive study of the early hypopharyngeal microbiota in 695 healthy infants at 1 week, 1 month, and 3 months of age.

We show that 63% of the hypopharyngeal microbiota at 3 months is from OTUs present already at 1 week. This strongly indicates that the airways of healthy individuals are colonized by bacteria and that very early colonization is important for the formation of the microbiota later in life. At the same time, the pattern of initial colonization by *Staphylococcus*, followed by better adapted colonizers (e.g., *Streptococcus*, *Moraxella)*, is similar to how the gut microbiota is colonized in early life [2]. Studies of the airway microbiota in infants have mainly investigated the nasopharyngeal region. Biesbroek et al*.* have presented two studies of the nasopharyngeal microbiota from 6 weeks to 6 or 24 months in which they have found early presence of *Staphylococcus*, followed by later dominance of *Moraxella* and *Corynebacterium* [21, 22]. We found *Staphylococcus* dominated at 1 week but rapidly declined over the next 3 months, indicating a general trend that it is an initial colonizer of the upper airways, which only has a significant presence within the first 6 months of life. Furthermore, the high prevalence of *Streptococcus* and *Moraxella* is similar to results from studies of the nasal swabs collected in Switzerland [23].

We present five pneumotypes, which are defined using a robust methodology inspired by the approach used to identify gut enterotypes [4] and characterized them with regard to composition, indicator OTUs, and development over time. We found that 9.4% of the infants had the same pneumotype at all three time-points. Bosch et al*.* found similar data studying nasal swabs collected within the first 6 months of life, describing a cluttered pattern that, when studied in depth, present clear microbial succession patterns [24].

The presence of pneumotypes and their temporal non-random changes indicates that non-random drivers shape the hypopharyngeal microbiota. We found that having siblings had the most significant impact on the hypopharyngeal microbiota, with only children more likely to present pneumotype I at 3 months of age. Furthermore, we found that delivery method, antibiotic use, and breastfeeding were significantly correlated with the microbiota at 1 month, but not at 1 week. This indicates that these variables do not change the early dominance by *Staphylococcus*, while still being important for the development of the hypopharyngeal microbiota when *Staphylococcus* no longer dominates.

Through studies of fecal samples as indicators of gut microbiota in early life, the gut microbiota was shown to affect development of the immune and lifestyle disorders such as obesity and diabetes [8, 25–27]. Similarly, studies of the upper airway microbiota, as an indicator of the lower airway microbiota, show correlation with disease development later in life [17, 28]. The vast surface areas of the airways provide intimate contact between microbiota and the immune system. Therefore, the early hypopharyngeal microbiota may similarly be studied as surrogate for the lower airway microbiota’s immune modulation ability, e.g., leading to increased airway inflammation, which has been indicated using murine models [18] and observed in children linking early life bacterial colonization to asthma [16].

Published data have shown that an infant’s nasopharyngeal microbiota dissimilarity increases over time while still having lower within-subject, than between-subject, dissimilarity [23]. We identified a similar pattern, the weighted UniFrac distances suggested that the bacterial community present at 1 week might not be predictive of the microbiota at 3 months; however, we could follow the development when including the 1-month sample. This indicates that the time span between 1 week and 3 months may be too long when analyzing the initial colonization of the hypopharynx, and that it would be prudent to increase the sampling frequency in future studies.

To appreciate the results of this study, the limitations of our study should be taken into account. The cohort we have studied was established in order to study more than just the development of the hypopharyngeal microbiota. Therefore, the cohort did not have as strict exclusion criteria as other studies, i.e. we have included infants born earlier than week 36 of gestation (3%) and did not excluded infants if they had been treated with antibiotics prior to sampling (5%). The cohort is generally representative for Denmark, with a slight skew towards maternal asthma and atopic disease, as well as socioeconomic factors (employment and household income) [19]. Furthermore, we acknowledge that reported associations to the clinical exposures are unadjusted, and future work will be needed to identify interactions between various influences on microbiota. This study was designed for classical culturing and identification of microbiota, without the rigorous focus on negative controls in all steps of the process we lack negative controls taken during sampling. Lastly, we have shown that samples with the lowest biomass did not have a higher richness than our DNA extraction negative controls and with SDI different from, but close to, the DNA extraction negative controls.

The strength of this study is the size of the cohort studied, with samples from 695 infants and most sampled at all time-points. Furthermore, the extensive focus on confirmation and verification has provided extensive and high quality metadata.

## Conclusions

In summary, we found that healthy infant airways are colonized by a core microbiota, which contains the majority of the hypopharyngeal microbiota. Our findings demonstrate that there is an overall time-dependent development of the hypopharyngeal microbiota, i.e., the decrease of *Staphylococcus* and increase of genera normally present in the airways, e.g., *Streptococcus*, *Moraxella*, and *Haemophilus* [11, 17, 29], leading to the formation of a microbiota community which can be separated into specific pneumotypes. Furthermore, we found that having siblings had the most significant impact on which pneumotype an infant would present, as it was seen that infants with siblings were less likely to harbor a hypopharyngeal microbiota dominated by *Staphylococcus*.

Our findings indicate that formation and development patterns of the hypopharyngeal microbiota share similarities with gut microbiota. We report that the very early colonization of the hypopharynx is of important for hypopharyngeal microbiota development. Furthermore, as others have shown that airway microbiota composition is related to development of asthmatic disease, indicating that the very early microbiota might influence the risk of asthma later in life.

## Methods

### Study population and sample collection

COPSAC_2010_ is an ongoing Danish cohort study of 700 unselected children and their families followed prospectively from pregnancy week 24 in a protocol designed from the first COPSAC birth cohort (COPSAC_2000_) [30]. Exclusion criteria were gestational age below week 26, maternal daily intake of more than 600 IU vitamin D during pregnancy, or having any endocrine, heart, or kidney disorders [19].

The hypopharyngeal microbiota was sampled from healthy infants during clinical visits at 1 week, 1 month, and 3 months of age. Hypopharyngeal aspirates were collected with a soft suction catheter passed through the nose into the hypopharynx as previously described in detail [19]. If the infants suffered from acute airway symptoms during their clinical visit, or if their parents reported wheezing or coughing in the days before or after sampling, the samples were classified as “acute” and excluded from this study. Antibiotic use was recorded as a categorical variable (yes/no) of whether the infant had received antibiotics at birth or at any other time before sampling. Only breastfeeding was recorded as a categorical variable (yes/no) of whether the infant had been introduced to any other food before sampling. In total 1988 samples were collected and initially included in the study (Additional file 1: Table S2). The aspirates were diluted in 1-ml sterile 0.9% NaCl and transported to the microbiological laboratory at Statens Serum Institut, Copenhagen, Denmark. Here, the samples were distributed into 150-μl aliquots and stored at −80 °C.

### DNA extraction and 16S amplicon sequencing

Genomic DNA was extracted using the PowerMag® Soil DNA Isolation Kit optimized for epMotion® (MO-BIO Laboratories, Inc., Carlsberg, CA, USA) using the epMotion® robotic platform model (Eppendorf) under manufacturer’s protocol. One hundred fifty microliters was used from each sample. At least one DNA extraction negative controls was included in each 96-well plate, by adding 150 μl of molecular grade water (Sigma-Aldrich, Merck, Germany) instead of a sample. DNA concentrations were determined using the Quant-iT™ PicoGreen® quantification system (Life Technologies, CA, US). Extracted DNA was stored at −20 °C.

The 16S rRNA gene amplification procedure was divided into two PCR steps: first, amplification of the hypervariable V4 region of the 16S rRNA gene, using the modified broad range primers 515F (5′-GTGCCAGCMGCCGCGGTAA-3′) and 806R (5′-GGACTACHVGGGTWTCTAAT-3′) [31–33], then sequencing primers and adaptors were added to the amplicon products in the second PCR step. The amplification products were then purified with Agencourt AMPure XP Beads (Beckman Coulter Genomics, MA, USA), pooled equimolar, concentrated using the DNA Clean & Concentrator™-5 Kit (Zymo Research, Irvine, CA, USA), and the concentrations were then determined using the Quant-iT™ High-Sensitivity DNA Assay Kit (Life Technologies). Paired-end sequencing, of up to 192 samples, was performed on the Illumina MiSeq System (Illumina Inc., CA, USA), including 1.0% PhiX as internal control. All reagents used were from the MiSeq Reagent Kits v2 (Illumina Inc., CA, USA). All details have been included in Additional file 1: Method S1.

### Bioinformatics analysis

Fastq files demultiplexed by the MiSeq Controller Software were trimmed for amplification primers, diversity spacers, and sequencing adapters (biopieces [34]), mate-paired and quality filtered (usearch v7.0.1090 [35], parameter: −maxee 0.5). UPARSE [36] was used for OTU clustering as recommended (97% similarity cut off), and for removing singletons after dereplication. Chimera checking was performed with usearch against the gold database [37] as recommended. Representative sequences were classified (Mothur v.1.25.0 [38], wang() function at 0.8 confidence threshold). Qiime wrappers for PyNAST [39], FastTree [40], and filter_alignment.py [41] were used to construct a phylogenetic tree. Alignments were built against the 2011 version of Greengenes [42] (parameters: --allowed_gap_frac 0.999999 and --threshold 3.0).

The rarefaction curves (Additional file 1: Figure S3) show the observed richness and the SDI as functions of count of sequences. SDI curves reach asymptotes with 1000 sequences; based on this, all samples with less than 2000 sequences were excluded. Additionally, two samples were excluded because of unusually high diversity; their SDI was more than 5 standard deviations higher than the mean. We included 1788 samples containing an average of 52,749 sequences per sample, representing 3715 distinct OTUs. To avoid bias due to sampling depth, we removed the difference by randomly subsampling the OTU table at even sequencing depth of 2000 observations. All further post-analyses were based on the even OTU table.

### Low biomass samples

**Fig. 5:**
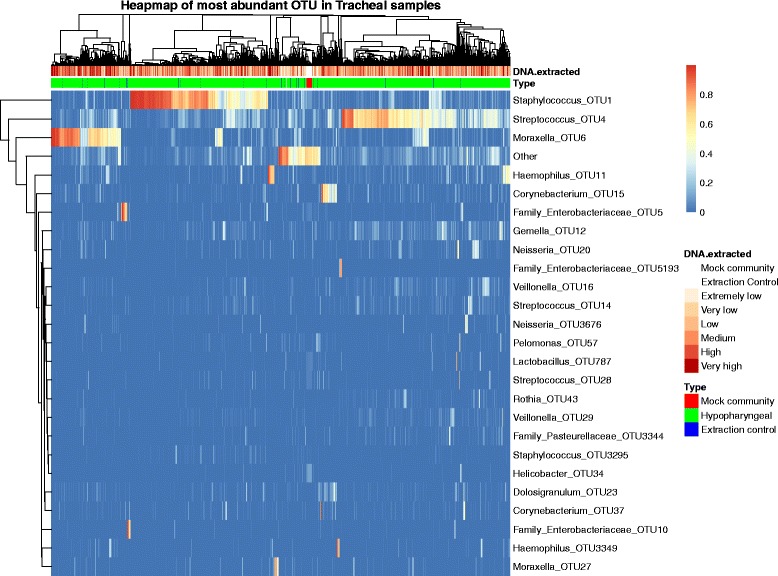
Heatmap of the 25 most abundant OTUs. The heatmap is annotated to show the amount of DNA extracted from each sample as well as the type of sample. Both samples and OTUs are clustered based on their Euclidian distances. The heatmap is colored by the log transformed relative abundance of each OTU within the samples

For comparison of control samples (mock communities and DNA extraction negative controls) and hypopharyngeal samples, we separated the hypopharyngeal samples into six groups based on the amount of DNA measured after DNA extraction (Extremely﻿ low:<0.1 ng/μl, very low: <0.33 ng/μl, low: <0.8 ng/μl, medium: <1.79 ng/μl, high: <4.58 ng/μl, very high: ≥4.58 ng/μl). We used analysis of variance, followed by Tukey multiple comparisons of means to determine which groups were significant different from each other. To create the following heatmap (Fig. 5), we used the *R* function “pheatmap” (R-package “pheatmap”) [43].

### Statistical analysis

For data treatment and analysis we used the open source statistical program “R”[44], predominantly the R-package “phyloseq” [45]. Wilcoxon rank sum test with continuity correction of difference in core OTUs abundance was achieved using function “wilcox.test” (*R*-package “stats”). Variation in continuous variables between groups was tested by analysis of variance, using the function “aov” to create the model, testing the overall variance using the function “anova” and the variance between individual groups with the function “TukeyHSD” (all three from *R*-package “stats”). For variation in count values of categorical variables (i.e., breastfeeding, antibiotic use, gender) between groups, we used Pearson’s *χ* ^2^ test of the significance (function “chisq.test,” *R*-package “stats”). The statistical significance of the difference in distances to each infant’s own sample and other infant’s samples was achieved using permutation, by randomly assigning which column contained the within sample.

### Characterization of pneumotypes

Clustering analysis was performed using partitioning around medoid (PAM) clustering [46]. The Silhouette index, using both weighted UniFrac distances and Jensen–Shannon divergence, showed that 5 clusters were optimal (0.35 and 0.39, respectively) [47]; subsequent clustering was based on weighted UniFrac distances. NMDS ordination was performed using the function “metaMDS” (*R*-package “vegan” [48]) and the weighted UniFrac distances. Indicator OTUs were identified using function “multipatt” (func = “indVal.g,” *R*-package “indicspecies” [20]).

## Additional files

Additional file 1: Figure S1. Alpha diversity comparison of hypopharyngeal samples, DNA extraction negative controls, and mock communities. The four boxplots showing the number of sequencing reads, the observed richness, the SDI, and the evenness of each sample. The samples have been separated by type and by concentration of extracted DNA, for the hypopharyngeal samples. The boxes contain all samples from the first to third quartile, with a line representing the median sample, and the hinges extend to the last sample within 1.5 times the range of the box. **Figure S3.** Rarefaction curves showed a trend of an increasing diversity over time. Rarefaction curves were calculated for both observed richness and SDI, grouped by time-point and with bars indicating sd. The observed richness did not reach saturation before 15,000 reads, whereas the SDI reaches a maximum after 1000 reads. **Table S1.** Alpha diversity by season. **Table S2.** Sample overview. **Table S3.** Most common OTUs in the core microbiota. **Table S4.** NMDS coordinates for the six samples which were excluded from the plots in Fig. 2. **Table S5.** Indicator values of the most significant indicator OTUs for each pneumotype. **Table S6.** Distribution of samples between the five pneumotypes at each time-point. **Table S7.** Number of infants presenting the same pneumotype continuously. **Table S8.** Abundance of core microbiota over time. **Method S1.** 16S rRNA gene amplification procedure with all details needed for reproduction. (DOCX 382 kb)
Additional file 2: Figure S2. Relative abundance of hypopharyngeal microbiota taxa over time. Krona plot showing the overall composition of the hypopharyngeal microbiota in all samples or at each time-point separately. This figure is interactive, and in the upper left corner the settings can be chosen; “Select dataset” to show data for 1 week, 1 month, 3 months, or all samples. “Max depth” sets to which taxonomic level the data is aggregated, *1* phylum, *2* class, *3* order, *4* family, *5* genus, *6* OTU. “Collapse” simplifies the chart by collapsing “redundant” wedges that are entirely composed of another wedge. “Snapshot” creates an svg image of the current display, and “Link” creates a link, including the current customized view. (HTML 403 kb)

## References

[1] Vael C, Desager K (2009). The importance of the development of the intestinal microbiota in infancy. Curr Opin Pediatr.

[2] Stark PL, Lee A (1982). The microbial ecology of the large bowel of breast-fed and formula-fed infants during the first year of life. J Med Microbiol.

[3] Palmer C, Bik EM, DiGiulio DB, Relman DA, Brown PO (2007). Development of the human infant intestinal microbiota. PLoS Biol.

[4] Arumugam M, Raes J, Pelletier E, Le Paslier D, Yamada T, Mende DR, Fernandes GR, Tap J, Bruls T, Batto J-M, Bertalan M, Borruel N, Casellas F, Fernandez L, Gautier L, Hansen T, Hattori M, Hayashi T, Kleerebezem M, Kurokawa K, Leclerc M, Levenez F, Manichanh C, Nielsen HB, Nielsen T, Pons N, Poulain J, Qin J, Sicheritz-Ponten T, Tims S (2011). Enterotypes of the human gut microbiome. Nature.

[5] Maynard CL, Elson CO, Hatton RD, Weaver CT (2012). Reciprocal interactions of the intestinal microbiota and immune system. Nature.

[6] Giongo A, Gano KA, Crabb DB, Mukherjee N, Novelo LL, Casella G, Drew JC, Ilonen J, Knip M, Hyöty H, Veijola R, Simell T, Simell O, Neu J, Wasserfall CH, Schatz D, Atkinson MA, Triplett EW (2011). Toward defining the autoimmune microbiome for type 1 diabetes. ISME J.

[7] Morgan XC, Tickle TL, Sokol H, Gevers D, Devaney KL, Ward DV, Reyes JA, Shah SA, LeLeiko N, Snapper SB, Bousvaros A, Korzenik J, Sands BE, Xavier RJ, Huttenhower C (2012). Dysfunction of the intestinal microbiome in inflammatory bowel disease and treatment. Genome Biol.

[8] Bisgaard H, Li N, Bonnelykke K, Chawes BLK, Skov T, Paludan-Müller G, Stokholm J, Smith B, Krogfelt KA (2011). Reduced diversity of the intestinal microbiota during infancy is associated with increased risk of allergic disease at school age. J Allergy Clin Immunol.

[9] Helander HF, Fändriks L (2014). Surface area of the digestive tract—revisited. Scand J Gastroenterol.

[10] Hasleton PS (1972). The internal surface area of the adult human lung. J Anat.

[11] Hilty M, Burke C, Pedro H, Cardenas P, Bush A, Bossley C, Davies J, Ervine A, Poulter L, Pachter L, Moffatt MF, Cookson WOC (2010). Disordered microbial communities in asthmatic airways. PLoS One.

[12] Huang YJ, Kim E, Cox MJ, Brodie EL, Brown R, Wiener-Kronish JP, Lynch SV (2010). A persistent and diverse airway microbiota present during chronic obstructive pulmonary disease exacerbations. OMICS.

[13] Coburn B, Wang PW, Diaz Caballero J, Clark ST, Brahma V, Donaldson S, Zhang Y, Surendra A, Gong Y, Elizabeth Tullis D, Yau YCW, Waters VJ, Hwang DM, Guttman DS (2015). Lung microbiota across age and disease stage in cystic fibrosis. Sci Rep.

[14] Charlson ES, Bittinger K, Haas AR, Fitzgerald AS, Frank I, Yadav A, Bushman FD, Collman RG (2011). Topographical continuity of bacterial populations in the healthy human respiratory tract. Am J Respir Crit Care Med.

[15] Bassis CM, Erb-Downward JR, Dickson RP, Freeman CM, Schmidt TM, Young VB, Beck JM, Curtis JL, Huffnagle GB (2015). Analysis of the upper respiratory tract microbiotas as the source of the lung and gastric microbiotas in healthy individuals. MBio.

[16] Bisgaard H, Hermansen MN, Buchvald F, Loland L, Halkjaer LB, Bønnelykke K, Brasholt M, Heltberg A, Vissing NH, Thorsen SV, Stage M, Pipper CB (2007). Childhood asthma after bacterial colonization of the airway in neonates. N Engl J Med.

[17] Teo SM, Mok D, Pham K, Kusel M, Serralha M, Troy N, Holt BJ, Hales BJ, Walker ML, Hollams E, Bochkov YA, Grindle K, Johnston SL, Gern JE, Sly PD, Holt PG, Holt KE, Inouye M (2015). The infant nasopharyngeal microbiome impacts severity of lower respiratory infection and risk of asthma development. Cell Host Microbe.

[18] Gollwitzer ES, Saglani S, Trompette A, Yadava K, Sherburn R, McCoy KD, Nicod LP, Lloyd CM, Marsland BJ (2014). Lung microbiota promotes tolerance to allergens in neonates via PD-L1. Nat Med.

[19] Bisgaard H, Vissing NH, Carson CG, Bischoff AL, Følsgaard NV, Kreiner-Møller E, Chawes BLK, Stokholm J, Pedersen L, Bjarnadóttir E, Thysen AH, Nilsson E, Mortensen LJ, Olsen SF, Schjørring S, Krogfelt KA, Lauritzen L, Brix S, Bønnelykke K (2013). Deep phenotyping of the unselected COPSAC2010 birth cohort study. Clin Exp Allergy.

[20] De Cáceres M, Legendre P (2009). Associations between species and groups of sites: indices and statistical inference. Ecology.

[21] Biesbroek G, Bosch AATM, Wang X, Keijser BJF, Veenhoven RH, Sanders EAM, Bogaert D (2014). The impact of breastfeeding on nasopharyngeal microbial communities in infants. Am J Respir Crit Care Med.

[22] Biesbroek G, Tsivtsivadze E, Sanders EAM, Montijn R, Veenhoven RH, Keijser BJF, Bogaert D (2014). Early respiratory microbiota composition determines bacterial succession patterns and respiratory health in children. Am J Respir Crit Care Med.

[23] Mika M, Mack I, Korten I, Qi W, Aebi S, Frey U, Latzin P, Hilty M (2015). Dynamics of the nasal microbiota in infancy: a prospective cohort study. J Allergy Clin Immunol.

[24] Bosch AATM, Levin E, van Houten MA, Hasrat R, Kalkman G, Biesbroek G, de Steenhuijsen Piters WAA, de Groot P-KCM, Pernet P, Keijser BJF, Sanders EAM, Bogaert D (2016). Development of upper respiratory tract microbiota in infancy is affected by mode of delivery. EBioMedicine.

[25] Kalliomäki M, Collado MC, Salminen S, Isolauri E (2008). Early differences in fecal microbiota composition in children may predict overweight. Am J Clin Nutr.

[26] Abrahamsson TR, Jakobsson HE, Andersson AF, Björkstén B, Engstrand L, Jenmalm MC (2012). Low diversity of the gut microbiota in infants with atopic eczema. J Allergy Clin Immunol.

[27] Hartstra AV, Bouter KEC, Bäckhed F, Nieuwdorp M (2015). Insights into the role of the microbiome in obesity and type 2 diabetes. Diabetes Care.

[28] Bisgaard H, Hermansen MN, Bønnelykke K, Stokholm J, Baty F, Skytt NL, Aniscenko J, Kebadze T, Johnston SL (2010). Association of bacteria and viruses with wheezy episodes in young children: prospective birth cohort study. BMJ.

[29] Marsland BJ, Yadava K, Nicod LP (2013). The airway microbiome and disease. Chest.

[30] Bisgaard H (2004). The Copenhagen Prospective Study on Asthma in Childhood (COPSAC): design, rationale, and baseline data from a longitudinal birth cohort study. Ann Allergy Asthma Immunol.

[31] Neefs JM, De Wachter R (1990). A proposal for the secondary structure of a variable area of eukaryotic small ribosomal subunit RNA involving the existence of a pseudoknot. Nucleic Acids Res.

[32] Yu Y, Lee C, Kim J, Hwang S (2005). Group-specific primer and probe sets to detect methanogenic communities using quantitative real-time polymerase chain reaction. Biotechnol Bioeng.

[33] Sundberg C, Al-Soud WA, Larsson M, Alm E, Yekta SS, Svensson BH, Sørensen SJ, Karlsson A (2013). 454 pyrosequencing analyses of bacterial and archaeal richness in 21 full-scale biogas digesters. FEMS Microbiol. Ecol..

[34] Hansen MA. Biopieces [Internet]. 2015. Available from: www.biopieces.org. Accessed Mar 2016.

[35] Edgar RC (2010). Search and clustering orders of magnitude faster than BLAST. Bioinformatics.

[36] Edgar RC (2013). UPARSE: highly accurate OTU sequences from microbial amplicon reads. Nat Methods.

[37] Haas BJ, Gevers D, Earl AM, Feldgarden M, Ward DV, Giannoukos G, Ciulla D, Tabbaa D, Highlander SK, Sodergren E, Methé B, DeSantis TZ, Petrosino JF, Knight R, Birren BW, Human Microbiome Consortium (2011). Chimeric 16S rRNA sequence formation and detection in Sanger and 454-pyrosequenced PCR amplicons. Genome Res.

[38] Schloss PD, Westcott SL, Ryabin T, Hall JR, Hartmann M, Hollister EB, Lesniewski RA, Oakley BB, Parks DH, Robinson CJ, Sahl JW, Stres B, Thallinger GG, Van Horn DJ, Weber CF (2009). Introducing mothur: open-source, platform-independent, community-supported software for describing and comparing microbial communities. Appl Environ Microbiol.

[39] Caporaso JG, Bittinger K, Bushman FD, DeSantis TZ, Andersen GL, Knight R (2010). PyNAST: a flexible tool for aligning sequences to a template alignment. Bioinformatics.

[40] Price MN, Dehal PS, Arkin AP (2009). FastTree: computing large minimum evolution trees with profiles instead of a distance matrix. Mol Biol Evol.

[41] Caporaso JG, Kuczynski J, Stombaugh J, Bittinger K, Bushman FD, Costello EK, Fierer N, Peña AG, Goodrich JK, Gordon JI, Huttley GA, Kelley ST, Knights D, Koenig JE, Ley RE, Lozupone CA, McDonald D, Muegge BD, Pirrung M, Reeder J, Sevinsky JR, Turnbaugh PJ, Walters WA, Widmann J, Yatsunenko T, Zaneveld J, Knight R (2010). QIIME allows analysis of high-throughput community sequencing data. Nat Methods.

[42] McDonald D, Price MN, Goodrich J, Nawrocki EP, DeSantis TZ, Probst A, Andersen GL, Knight R, Hugenholtz P (2012). An improved Greengenes taxonomy with explicit ranks for ecological and evolutionary analyses of bacteria and archaea. ISME J.

[43] Kolde R. pheatmap: Pretty Heatmaps. 2015.

[44] R Core Team. R (2015). A language and environment for statistical computing.

[45] McMurdie PJ, Holmes S. phyloseq: an R package for reproducible interactive analysis and graphics of microbiome census data. Watson M, editor. *PLoS One* 2013; 8: e61217. 10.1371/journal.pone.0061217 PMC3632530 23630581

[46] Maechler M, Rousseeuw P, Struyf A, Hubert M, Hornik K. cluster: Cluster analysis basics and extensions. 2015

[47] Rousseeuw PJ (1987). Silhouettes: a graphical aid to the interpretation and validation of cluster analysis. J Comput Appl Math.

[48] Oksanen J, Blanchet FG, Kindt R, Legendre P, Minchin PR, O’Hara RB, Simpson GL, Solymos P, Stevens MHH, Wagner H. vegan: Community Ecology Package. 2015

