# Supplementary material for: The developing hypopharyngeal microbiota in early life
Source: Microbiome. 2016 Dec 30;4:70. doi: 10.1186/s40168-016-0215-9 (PMC5203717; doi:10.1186/s40168-016-0215-9)
Supplement: Additional file 1: Figure S1. — Alpha diversity comparison of hypopharyngeal samples, DNA extraction negative controls, and mock communities. The four boxplots showing the number of sequencing reads, the observed richness, the SDI, and the evenness of each sample. The samples have been separated by type and by concentration of extracted DNA, for the hypopharyngeal samples. The boxes contain all samples from the first to third quartile, with a line representing the median sample, and the hinges extend to the last sample within 1.5 times the range of the box. Figure S3. Rarefaction curves showed a trend of an increasing diversity over time. Rarefaction curves were calculated for both observed richness and SDI, grouped by time-point and with bars indicating sd. The observed richness did not reach saturation before 15,000 reads, whereas the SDI reaches a maximum after 1000 reads. Table S1. Alpha diversity by season. Table S2. Sample overview. Table S3. Most common OTUs in the core microbiota. Table S4. NMDS coordinates for the six samples which were excluded from the plots in Fig. 2. Table S5. Indicator values of the most significant indicator OTUs for each pneumotype. Table S6. Distribution of samples between the five pneumotypes at each time-point. Table S7. Number of infants presenting the same pneumotype continuously. Table S8. Abundance of core microbiota over time. Method S1. 16S rRNA gene amplification procedure with all details needed for reproduction. (DOCX 382 kb) [file 40168_2016_215_MOESM1_ESM.docx]

**Supplementary Data**

**Figure S1. Alpha diversity comparison of hypopharyngeal samples, DNA extraction negative controls and Mock communities.** The four boxplots show a) the number of sequencing reads, b) the observed richness, c) the Shannon Diversity Index, and d) the evenness of each sample. The samples have been separated by type and by concentration of extracted DNA, for the hypopharyngeal samples. The boxes contains all samples from the first to third quartile, with a line representing the median sample, and the hinges extend to the last sample with 1.5 time the range of the box.

**Figure S3. Rarefaction curves showed a trend of an increasing diversity over time.** Rarefaction curves were calculated for both observed richness and Shannon diversity, grouped by time-point and with bars indicating sd. The observed richness did not reach saturation before 15,000 reads, whereas the Shannon diversity reaches a maximum after 1,000 reads.

**Table S1: Alpha diversity by time-point and season**

|  | Observed richness | | | | | | | | |
| --- | --- | --- | --- | --- | --- | --- | --- | --- | --- |
| Season | One week | | | One month | | | Three months | | |
|  | mean | sd | S | mean | sd | S | mean | sd | S |
| Spring | 24.30 | ±13.91 | ^a^ | 29.64 | ±16.83 | ^a^ | 33.95 | ±12.74 | ^a^ |
| Summer | 19.71 | ±8.72 | ^b^ | 22.63 | ±8.80 | ^b^ | 30.19 | ±12.48 | ^b^ |
| Fall | 22.65 | ±9.17 | ^ab^ | 24.29 | ±9.95 | ^bc^ | 30.17 | ±12.73 | ^ab^ |
| Winter | 22.15 | ±8.06 | ^ab^ | 26.50 | ±10.30 | ^ac^ | 31.61 | ±12.57 | ^ab^ |
|  | Shannon Diversity Index | | | | | | | | |
| Season | One week | | | One month | | | Three months | | |
|  | mean | sd | S | mean | sd | S | mean | sd | S |
| Spring | 1.08 | ±0.57 | ^a^ | 1.35 | ±0.60 | ^a^ | 1.49 | ±0.60 | ^a^ |
| Summer | 0.95 | ±0.59 | ^a^ | 1.09 | ±0.56 | ^b^ | 1.39 | ±0.57 | ^a^ |
| Fall | 1.08 | ±0.67 | ^a^ | 1.19 | ±0.57 | ^ab^ | 1.42 | ±0.61 | ^a^ |
| Winter | 1.16 | ±0.61 | ^a^ | 1.34 | ±0.55 | ^a^ | 1.40 | ±0.58 | ^a^ |

Overview of alpha diversity by season. Spring: March, April, and May. Summer: June, July, and August. Fall: September, October, and November. Winter: December, January, and February. The columns S marks the significant different samples (samples with the same letter are not significantly different from each other). The color in the mean column cells is read for the highest value and blue for the lowest value. The color scale is calculated for each time-point and measurement separately.

**Table S2. Sample overview.**

| Step | Samples | Reason removed | 3 samples | 2 samples | 1 sample |
| --- | --- | --- | --- | --- | --- |
| Samples taken | 1998 |  | 618 | 66 | 12 |
| Samples received | 1986 | Lost during transport  (12 samples) | 607 | 76 | 13 |
| DNA extraction | 1970 | Samples dropped  (16 samples) | 592 | 90 | 14 |
| Sequencing | 1946 | No sequences from sample  (24 samples) | 569 | 112 | 15 |
| Removal of unwanted samples | 1944 | Unusual high alpha-diversity  (2 samples) | 567 | 114 | 15 |
|  | 1788 | Less than 2000 reads  (156 samples) | 438 | 217 | 40 |

Overview of samples in this study and where in the workflow samples were removed. ^+^The number of infants from which all three samples are included.

**Table S3.** Most common OTUs in the core microbiota.

| OTU | infants (%) |
| --- | --- |
| Streptococcus_OTU4 | 96.3% |
| Staphylococcus_OTU1 | 80.5% |
| Moraxella_OTU6 | 60.4% |
| Gemella_OTU12 | 57.9% |
| Streptococcus_OTU3874 | 52.2% |
| Streptococcus_OTU1956 | 36.4% |
| Haemophilus_OTU11 | 33.4% |
| Corynebacterium_OTU15 | 30.9% |
| Staphylococcus_OTU3678 | 24.5% |
| Streptococcus_OTU14 | 18.1% |

Shown as the percentage of infants having this OTU as part of their core microbiota.

**Table S4**. **NMDS coordinates for the six samples which were excluded from the plots.**

| Sample | NMDS1 | NMDS2 | NMDS3 | NMDS4 | Time | Pneumotype |
| --- | --- | --- | --- | --- | --- | --- |
| 1385.B | -0.1259 | 0.2381 | 0.0632 | 0.2783 | 1 month | Pneumotype I |
| 1558.A | NR | NR | -0.0633 | 0.2196 | 1 week | Pneumotype V |
| 1186.A | NR | NR | -0.3741 | 0.6181 | 1 week | Pneumotype IV |
| 1336.A | NR | NR | 0.0180 | 0.2666 | 1 week | Pneumotype V |
| 1344.A | 0.4400 | 0.1108 | 0.0810 | 0.4906 | 1 week | Pneumotype V |
| 1085.C | 0.4171 | 0.0290 | NR | NR | 3 months | Pneumotype III |
| 1529.C | 0.2849 | -0.2595 | 0.0739 | 0.3748 | 3 months | Pneumotype V |

NR indicate that the sample was not removed in that plot.

**Table S5.** **Indicator values for the most significant indicator OTUs for each pneumotype.**

| Pneumotype | OTU | All samples | | | 1 week | | | 1 month | | | 3 months | | |
| --- | --- | --- | --- | --- | --- | --- | --- | --- | --- | --- | --- | --- | --- |
|  |  | A^a^ | B^b^ | Stat^c^ | A^a^ | B^b^ | Stat^c^ | A^a^ | B^b^ | Stat^c^ | A^a^ | B^b^ | Stat^c^ |
| Pneumotype  I | Staphylococcus_OTU1 | 0.75 | 1.00 | 0.87 | 0.64 | 1.00 | 0.80 | 0.75 | 0.99 | 0.86 | 0.82 | 1.00 | 0.90 |
|  | Staphylococcus_OTU3153 | 0.68 | 0.99 | 0.82 | 0.56 | 0.99 | 0.75 | 0.68 | 0.98 | 0.82 | 0.79 | 0.98 | 0.88 |
|  | Staphylococcus_OTU3678 | 0.66 | 0.99 | 0.81 | 0.53 | 1.00 | 0.72 | 0.76 | 0.99 | 0.86 | 0.77 | 0.98 | 0.87 |
|  | Staphylococcus_OTU3697 | 0.59 | 0.97 | 0.76 | 0.59 | 0.99 | 0.77 | 0.61 | 0.96 | 0.77 | 0.77 | 0.93 | 0.84 |
|  | Staphylococcus_OTU765 |  |  |  |  |  |  |  |  |  | 0.70 | 0.69 | 0.69 |
|  | Family_Staphylococcaceae_OTU4172 | 0.75 | 0.72 | 0.74 | 0.63 | 0.73 | 0.68 | 0.78 | 0.74 | 0.76 |  |  |  |
| Pneumotype II | Streptococcus_OTU1956 | 0.66 | 0.94 | 0.79 | 0.57 | 0.95 | 0.73 | 0.63 | 0.95 | 0.77 | 0.66 | 0.93 | 0.79 |
|  | Streptococcus_OTU4 | 0.53 | 1.00 | 0.73 | 0.62 | 1.00 | 0.79 |  |  |  | 0.52 | 1.00 | 0.72 |
|  | Streptococcus_OTU4044 | 0.59 | 0.70 | 0.64 | 0.63 | 0.78 | 0.70 | 0.57 | 0.79 | 0.67 |  |  |  |
|  | Streptococcus_OTU14 | 0.54 | 0.74 | 0.64 |  |  |  | 0.61 | 0.85 | 0.72 |  |  |  |
|  | Streptococcus_OTU3874 |  |  |  | 0.56 | 0.98 | 0.74 |  |  |  |  |  |  |
|  | Veillonella_OTU16 | 0.55 | 0.73 | 0.64 |  |  |  | 0.59 | 0.78 | 0.68 |  |  |  |
|  | Gemella_OTU12 |  |  |  | 0.52 | 0.88 | 0.68 |  |  |  | 0.50 | 0.94 | 0.69 |
| Pneumotype III | Moraxella_OTU6 | 0.67 | 0.95 | 0.80 | 0.70 | 0.92 | 0.80 | 0.67 | 0.95 | 0.80 | 0.63 | 0.96 | 0.78 |
| Pneumotype IV | Corynebacterium_OTU15 | 0.87 | 0.89 | 0.88 | 0.90 | 0.71 | 0.80 | 0.86 | 0.96 | 0.91 | 0.83 | 0.90 | 0.87 |
|  | Corynebacterium_OTU37 | 0.85 | 0.70 | 0.77 |  |  |  | 0.84 | 0.80 | 0.82 | 0.80 | 0.70 | 0.75 |
|  | Dolosigranulum_OTU23 | 0.73 | 0.71 | 0.72 |  |  |  | 0.69 | 0.75 | 0.72 | 0.82 | 0.80 | 0.81 |
| Pneumotype V | No indicator OTU found |  |  |  |  |  |  |  |  |  |  |  |  |

### ^a^The Positive predictive power of OTU as indicator for the given pneumotype (Specificity). ^b^The sensitivity of the OTU as indicator for the given pneumotype (Fidelity). ^c^The statistical value of OTU as indicator for the given pneumotype. The indicator OTUs were calculated for all samples together and for each of the three time-points separately

**Table S6.** **Distribution of samples between the five pneumotypes at each time-point.**

| Pneumotype | 1 week (544) | 1 month (621) | 3 months (622) |
| --- | --- | --- | --- |
| Pneumotype I | 297 (55%) | 138 (22%) | 54 (9%) |
| Pneumotype II | 85 (16%) | 204 (33%) | 199 (32%) |
| Pneumotype III | 50 (9%) | 87 (14%) | 161 (26%) |
| Pneumotype IV | 24 (4%) | 55 (9%) | 30 (5%) |
| Pneumotype V | 88 (16%) | 137 (22%) | 178 (29%) |

**Table S7. Number of infants presenting the same pneumotype continuously.**

| Pneumotype | Observed infants (%)^a^ | Random Infants (%)^b^ | Ratio (Observed/Random)^c^ |
| --- | --- | --- | --- |
| Pneumotype I | 12 (4.0%) | 5.7 (1.9%) | 2.09 |
| Pneumotype II | 14 (16.5%) | 8.9 (10.5%) | 1.57 |
| Pneumotype III | 4 (8.0%) | 1.8 (3.6%) | 2.21 |
| Pneumotype IV | 0 | 0.1 (0.4%) | - |
| Pneumotype V | 13 (14.8%) | 5.6 (6.3%) | 2.34 |

^a^The number of infants continuously having the same pneumotype in this study. ^b^The number of infants who would have had this pneumotype continuously, if the infants were randomly distributed between the pneumotypes. ^c^The ratio between infants who were observed to have the pneumotype continuously and the number expected by random distribution.

**Table S8.** **Abundance of core microbiota over time.**

|  | Continuous^a^ (%) | Changing^b^ (%) | P-value^c^ | Difference (%-points) |
| --- | --- | --- | --- | --- |
| All | 85.8 | 77.6 | 0.003 | 8.1 |
| 1 week | 91.2 | 89.2 | 0.369 | 2.0 |
| 1 month | 85.0 | 80.6 | 0.186 | 4.4 |
| 3 Months | 79.2 | 61.6 | 0.001 | 17.6 |

^a^ Median abundance of core microbiota of infants having 1 pneumotype continuously. ^b^ Median abundance of core microbiota in infants that changes pneumotype. ^c^ The P-values calculated using Wilcoxon rank sum test with continuity correction.

**Method S1. 16S rRNA gene amplification procedure.**

**Step 1.** Amplification was performed in 96-well microtiter plates with a reaction mixture consisting of 1X AccuPrime PCR Buffer II, 0.6 U AccuPrime Taq DNA Polymerase (Invitrogen, Life technologies, CA, US), 0.5 µM primer 515F, 0.5 µM primer 806R, 2.0 µl template DNA, and molecular grade water (Sigma-Aldrich, Merck, Germany) to a total volume of 20.0 µl per sample. Reactions were run in a 2720 thermal cycler (Applied Biosystems®, Life Technologies, CA, US) according to the following cycling program: 2 minutes of denaturation at 94°C, followed by 30 cycles of 20 seconds at 94°C (denaturing), 30 seconds at 56°C (annealing) and 40 seconds at 68°C (elongation), with a final extension at 68°C for 5 minutes. For each plate, a negative template-free control and a positive control containing 2.0 µl DNA from a known bacterial mock community (1.0 ng/µl; HM-782D, BEI Resources, VA, US) were included. The PCR products were quantified using the Quant-iT™ PicoGreen® quantification system (Life Technologies, CA, US) and samples with a PCR product concentration above 6.0 ng/µl were diluted to approximately 3.0-6.0 ng/µl prior to further analysis.

**Step 2.** Sequencing primers and adaptors were added to the amplicon products in the second PCR step as follows: 2.0 µl of the diluted amplicons were mixed with a reaction solution consisting of 1X AccuPrime PCR Buffer II, 0.6U AccuPrime Taq DNA Polymerase (Invitrogen, Life technologies, CA, US), 0.5 µM fusion forward and 0.5 µM fusion reverse primer, and molecular grade water (Sigma-Aldrich, Merck, Germany) (total volume 20 µl). The PCR was run according to the cycling program above except with a reduced cycling number of 15.

The amplification products were purified with Agencourt AMPure XP Beads (Beckman Coulter Genomics, MA, US) using 0.7X volume beads and quantified as described above. Equimolar amounts of the amplification products were pooled together in a single tube. The pooled DNA samples were concentrated using the DNA Clean & Concentrator™-5 Kit (Zymo Research, Irvine, CA, US), and the concentration were then determined using the Quant-iT™ High-Sensitivity DNA Assay Kit (Life Technologies). Amplicon sequencing was performed on the Illumina MiSeq System (Illumina Inc., CA, US). For each run, a 1.0% PhiX internal control was included. All reagents used were from the MiSeq Reagent Kits v2 (Illumina Inc., CA, US). Automated cluster generation and 250 paired-end sequencing with dual-index reads were performed. The sequencing output was generated as demultiplexed fastq-files for downstream analysis. Up to 192 samples were sequenced per run.
