# Supplementary material for: The developing hypopharyngeal microbiota in early life
Source: Microbiome. 2016 Dec 30;4:70. doi: 10.1186/s40168-016-0215-9 (PMC5203717; doi:10.1186/s40168-016-0215-9)
Supplement: Additional file 2: Figure S2. — Relative abundance of hypopharyngeal microbiota taxa over time. Krona plot showing the overall composition of the hypopharyngeal microbiota in all samples or at each time-point separately. This figure is interactive, and in the upper left corner the settings can be chosen; “Select dataset” to show data for 1 week, 1 month, 3 months, or all samples. “Max depth” sets to which taxonomic level the data is aggregated, 1 phylum, 2 class, 3 order, 4 family, 5 genus, 6 OTU. “Collapse” simplifies the chart by collapsing “redundant” wedges that are entirely composed of another wedge. “Snapshot” creates an svg image of the current display, and “Link” creates a link, including the current customized view. (HTML 403 kb) [file 40168_2016_215_MOESM2_ESM.html]

Javascript must be enabled to view this page.

members
magnitude
magnitudeUnassigned

One Week
One Month
Three Months
All Samples

1084106124046712442693568842

9011127793679558585

3397166817826847

3397166817826847

228848320

220611282

1102

0101

21900219

0011

059059

85619

0202

0066

0101

82010

018119

0011

018018

194766132

023335

022426

0099

552030

0404

1001

0011

0022

4004

001717

0101

0303

0303

0022

0022

1437859

037037

0011

140014

0077

0033

0033

170724

170017

170017

0077

825033

3125151217016338

0011

0011

0022

0022

0044

0044

0202

0202

78227107

78227107

3047148816876222

234070052224531590

234070052224531590

0033

0033

0011

0022

810184345477200

351239241082

0101

0022

0101

351219221078

0033

0011

0022

0151530

04711

0011

0101

0022

010515

775170536026082

0033

0033

121262210472881

121262210472881

0066

0235

0022

0044

0044

0011

0077

0011

0011

0055

0011

0011

0011

0066

0099

0022

0011

0011

0011

0112

0101

0022

0011

1023

0011

0033

0022

0022

0011

0033

0011

021214

1012

0022

0044

12085929092709

0033

2172847

0101

0088

0101

0011

0011

0011

0516

4602874

27245401662021432

22244561539720075

1001

0101

4026

5211165381

92153651106738

0303

0022

395251349

1102

1001

0033

0011

0011

1001

0617

0101

144248431281

0022

0099

1326

4004

0055

1001

35108262117328

0011

0141630

0022

0055

1001

02911

3893518872860

0112

4004

0101

4004

124732

201416

123393417

2002

1124

038182220

0022

0437

029144173

0022

4004

0133245

0101

021214

0213

0011

1102

0202

007373

340640

0022

310031

0044

3003

168412171317

052934

052934

052934

052934

002929

0101

0202

0101

0101

35515571008511997

2919254426548117

2919254426548117

2919254426548117

201012

201012

39304397

0044

39300393

9069554542315

9069554542315

0101

0101

0011

0011

366326125

0088

0202

1101425

0011

0011

159024

051051

0123

100010

1258175308

0112

105015

1152174291

16062576798

152046198

11509511

0011

7007

0011

0611980

017017

017017

1001

1001

001111

001111

1174143813974009

12200122

12200122

032032

0303

0303

0303

0303

0101

0202

027027

027027

027027

027027

027027

0202

0202

0202

0202

0202

1067

1067

1067

0066

0066

1001

1001

1001

131216

131216

131216

131216

131216

0011

0101

121114

614515602017

614515602017

4004

4004

4004

1001

3003

214515602013

214515602013

214505431995

214505431995

011718

011718

19986513873036

0202

0202

0202

0202

0101

0101

120012

120012

120012

120012

120012

0101

0101

0101

0101

0101

20022117438

20022117438

20022117438

20022117438

047047

1203

1001

1122033

101011

105015

542653

2002

0101

1102

1203

1001

4004

081081

0202

12731131

222024

0101

0202

0101

0066

1146

1001

1203

1001

0303

5479742686

5479742686

5479742686

5479742686

4004

0101

0303

0202

1001

0202

2406

4004

3688542495

440044

12400124

2305

2305

2305

2305

2305

333266265864

333266265864

333266265864

333266265864

710071

0101

19205215

0303

1001

280028

110011

0099

0404

0202

0325

0707

172019

1102

0134

0101

0202

144182164

0101

0303

0101

58013

5102071

002222

0202

0022

01680168

39012

020020

0617

0617

0617

0617

0202

0011

0404

2002

2002

2002

2002

2002

13500135

13500135

13500135

13500135

2002

13300133

28100281

28100281

28100281

28100281

28100281

48172490

48172490

48172490

48172490

150015

40112404

0202

2103

630063

0202

0101

54857110

54857110

54857110

54857110

0606

0303

0101

012122

0101

0101

1067

2002

0101

0101

027027

0101

2002

0202

0011

002020

03912

0033

0033

0033

0033

0033

5144499

5144499

5144499

5144499

5144499

044448

510051

619025

619025

619025

619025

619025

014014

65011

58356741764223899

58356741764223899

58356741764223899

27729331223615446

27729331223615446

001414

06312651328

21532315292067

1331320942420

4597665167537

0417268685

069057747

177276354

374217294

306274154068453

606167

5005

106162

297270951568162

332189224

0011

0011

0022

0152338

0022

313236

0148094

0213

004747

2020513238

2020513238

2020513238

2020513238

2020513238

02030203

0033

0077

0101

0033

201021

0011

0011

0011

0011

0011

0011

395718948668044197101

395718948668044197101

0066

0066

0066

0044

0022

386618732666445192432

1824549276

1741849241

1741849241

6208

225027

225027

6365108971828335545

500151

500151

434379401741129694

37011705452085

116466

15123461

283772137

40793821633508

5173124248

4868118234

340656271428023313

17141142

2403125296

501015

2353115281

140615276823615

140615276823615

1293270231

1931367901650

3926146346

39272995

1151079151209

4048

4048

081788

081788

232459484

232459484

006565

1001

22297121

00297297

0246

0246

0246

0033

0033

0033

190019

190019

190019

001010

0077

0077

0033

0033

27532230627

27030273

27030273

531930354

531930354

1101680206

0101

0101

2326590

2326590

650166

22131449

22131449

220123145

00123123

00123123

220022

220022

0044

0044

0044

285357314042967144642

49210493

49210493

280437313942966144148

25317752482

0011

651920075788534479

0033

9242356

952097

60123101284

002727

203675254434691107602

0112

5167494684

402648

5423793

211821

12811427269

0011

1464161

001515

0011

001414

1462646

1462646

278582194

278582194

006363

278519131

451781539

2013

2013

411618435

0022

0022

3816099

3816099

557466

490150

490150

56314

56314

1102

473142

402832

302528

1034

0022

0022

0011

0011

0707

1792156831036463

0101

0101

0022

0022

0033

0033

1792156730986457

411339591133

83011

163288915494070

310107120

14765113

61121891

43437345825

2002

033538

1332054

820284

820284

820082

0022

18312

18312

18312

1974903690

1974903690

75113

17400174

164852503

271781125

0303

0101

0202

251181117

203638

1157

110011

9104059

2002

0303

0303

2002

2002

2013

2013

2013

110011

110011

110011

4775689942039

048048

048048

048048

1024126169

232429

232429

0303

002424

2002

9982109

130031

130031

120021

0606

0101

0101

0101

0101

2351624275

47814948682840

47814948682840

1021789316

1021789316

20510767612042

010010

010667173

1135

0246

0101

0202

0044

0101

0178

2029296481779

0279

15915

15915

010313

0101

26319515473

2002

105244133

15617111338

0314

0314

0325

365039125

8123353

280634

280634

0055

280129

038038

038038

038038

595596361254

595596361254

595586331250

545316201205

526637

0077

0101

0011

0011

0112

0011

0011

2139803378595227121074551

582480916928325

582480916928325

58142446106668

58142446106668

58142446106668

1056510821657

1067

1056

0011

1001

1001

856510761649

012271193

2046

0033

64439981447

1383932511271929809

015116

015116

015116

0303

012113

1597038267

602331114

05611

0101

0404

0066

0101

0101

402428

001818

0055

2002

0011

2002

5617174

3428062

6519791

0066

0066

0011

0011

4004

4004

330033

330033

260026

260026

218020

0101

0101

57311170773714638

57011168773514604

0022

0022

93649661

10143144

83506517

1552740404222

2402448

2402448

5513113830209671

260834

011011

4415546542

70310

14315258416

0077

010010

0101

4896104626988640

302234

6006

6006

242228

242228

155142112673239

1225

1225

0022

0022

0022

0099

0055

0055

0044

092938

0112

0112

0101

0101

072835

38834408830

335339377

303235

32337342

353861422

350661417

3003

0202

012820

0189

0505

0505

0101

29011

4004

4004

4004

857487

826373

0011

701017

125255

0077

0077

0347

137139

037138

037138

1001

1001

442785511071

442785511071

11915111245

25054411715

125320

0303

5611067

0077

0066

5038

20015179430

507105111723

0202

0202

1440862

4596398620

4596398620

340539

340539

0011

0011

0011

132446513893178

132446513893178

52593225843

130233

07140147

5225083655

0404

2103

0101

538735778

538735778

0101

0101

5312762501057

26357179499

231433268

1023

1951139209

122519

467322492

467322492

467322492

0011

4004

2013

5731575

40405409

139365512563304

321445210094675

321445210094675

002626

002626

9481254731546

851273511229

577357187

402565130

22503274363013

001010

53900539

17113274262464

1607490

167052296289449031912372

2698378727102449208159

2698378727102449208159

1056

1056

6460132491540335112

205226547887041173041

8122949

49328341

2419134311910434954

49655668

023840

180875201466884136985

0134

151723

0022

0022

0022

151521

121013

121013

3058

1012

0011

2002

0033

930093

930093

1001

1001

920092

920092

01100110

01100110

01100110

1405

1405

1405

0202

0101

1102

924512545

15481487

15481487

15481487

8008

8008

8008

0193150

019019

019019

003131

003131

131235143509

131235143509

100010

100010

121235143499

121235143499

3003

3003

3003

3003

41317110061590

0202

0202

0101

0101

41316910061588

364244284

364244284

0415

0415

026026

026026

28185197

19010

0606

1001

2635180

69135105

201214

001717

671068

0066

9009

9009

0077

0044

0033

189136226

18336120

06100106

75012

2002

5409

0101

26321522806

011011

011011

160016

7007

7007

7007

9009

9009

9009

280733902039122106215

280733902039122106215

287414172873

65415

281409168858

0022

0022

2792296511159

209597

0077

442652

1405

1215330

36302288

224138515877

0033

275073837738297104181

8433488842075

212023

202022

202022

0099

201113

0101

0101

0101

110470177648304888593006

75053869397515349

0101

0101

74073830394315180

8166723863269

25512197

10331830451

011213

2720047

0606

8196203511790

554526684198632

5131028

300104134

7108

00128128

29521596

13477481

943711142

412126

412126

102965173779300913577657

758511171

387111120

414018

330033

96516166401292692555609

91006151509266281508796

745122174

405174165

1416193268

2046

869130207

5130129822005438166

0134

13093913622431

7142849

416189209

041115

12107309428

1264267441296

951024

1631853192

206693179

0022

032427

032427

349180233762

0101

349179233761

60257110795121086

0606

55048103

248222544696942

0055

68249263

185736712613485

3901261817358254

54158167

31826231852

0639

264543727257818121544

3137

3137

0112

0112

3025

3025

147558111947632342

183620245

220022

220022

7811089

82390121

82390121

112013

112013

8323494061587

4733493831205

4733493831205

354023377

347021368

7029

5005

5005

17211323308

7262053

7222049

0303

0101

0112

0112

165862253

165862253

0011

9009

9009

9009

6937142248

1936112167

1936112167

5013081

134907550890429944

118536827604324723

118536827604324723

47203198

47203198

80946522643538

80946522643538

1315836

1315836

28318426727

485205132822

485205132822

6161986

6161986

001010

001010

0178

0178

54211

561057

561057

11576290714817488821

11576290714817488821

0022

0022

16715615991922

001919

001919

11409289154655486878

24195330549

0123

052025

3514528713051

026564590

0111930

6256091

1213912821433

047579

614567251242

0033

7564185402849454598

8928147993918978

089241330

2810107317455628

0226183

5152242

04101105

018018

447211127

29011

29011

29011

29011

132135150

132135150

0112

131134148

131134148

49321313492055

0103646

010010

010010

003636

003636

003636

3781486541180

32742569938

101314

100010

0101

0033

31741566924

1001

1001

1001

509585230

011011

011011

0022

0022

0022

7929598706

0101

0101

0101

7928598705

0112

0101

0011

7927597703

7926597702

0101

005757

005757

005656

0011

0011

3626264

31825103446

134017

134017

134017

134017

134017

1001

121013

0303

0000

0000

0000

0000

0000

0000

48151982

48151982

48151982

48151982

011516

001313

0011

0101

0011

0404

0404

300030

300030

0808

0808

182424

143320

143320

143320

143320

143320

1102

112013

2002

0022

0011

8117847892675966302197681

89605279718621425

0303

0303

0202

0202

0101

0101

0011

89605276718521421

12459394992683

650065

650065

1001

1001

153220

1001

0202

0011

0101

140115

0112

0112

4004

1001

3003

0011

0011

839092

0101

0606

832085

5121229

1001

0011

211114

1001

0404

0101

0202

0101

0303

1001

1668872871340

0101

4111530

2133963

0213

1397282401107

110520126

0404

0202

0011

1113

47011

0404

0101

1001

3104

0101

901191961116

1102

1102

014014

014014

014014

4169163273

415938138

062228

0011

0202

415046

046450

001111

010125135

0108494

004141

0011

0011

0011

0011

0011

0011

117920426743895

790512571098

7765130911

061723

0246

5252151

0099

4136077

001616

5005

132070103

10186189

0202

30912

703126127

703126127

5106

20752877812

20752577809

0303

08220228

08220228

941403241521

0101

941402241520

0044

0044

0044

1677141541077199

048286

048286

1001

1001

0707

0606

0101

0101

0101

001212

001212

0224

0022

0202

2881854

0112

0112

2861650

1302556

0112

001818

0134

027128

0022

0101

1001

1107081

007070

1001

010010

26681331678

0101

26571114450

0022

04209213

1102

0202

0268

1379125835546191

011011

011011

131317

131317

465859814776733

275436

8008

8008

462359314736689

720072

316754411814892

1618

2103

0011

332237

013637

003737

0033

0101

0044

11165167

001717

13777261410

156191250597

4004

4004

4004

08917

0099

0088

0011

0808

7809517435415489082073400

2088754203913937391023005

5728236375

531836107

0808

53103598

0011

42640268

0707

42570261

184894383112363700931706

182774382100363613928487

2872164264

2754644421181

977724198

159378339060342460840898

354125814953107

6523056738

5152848

418325

4201337

3014

1522340

1081533

293979881414

1270226526910948337

0011

2529

052328

1153657716309360

2221034

68195320583

312520

48335366489415093

22257743043303

25962411962079

2356149228

5203992291148

032023

1309329252

0202

0214768

2120991403151

2120989393148

0213

12131213302246055921

42231883

42231883

1723049

1723049

91313

91313

1111931

1111931

1141314251729455

1108288341788169

5122542

282479691244

10891181461721846255

10891181461721846255

151016

190019

190019

221525137005428

221525137005428

221525137005428

936012954663028944

933612921658628843

0011

85733641374358

1102

665374

0202

005757

27447285

132529

2524362626438793

59418

0112

034034

0505

0011

58404102

145560

3014

1001

133723388

1102

1124

6311075248

1124

1719

0134

0011

5117

1856411260

1337

231117547895

7029

0202

1001

0011

0202

2614275243

11201203

2742253

0101

0033

4911113173

56011

0101

442248

0202

48824871267812431

1012

0033

0033

24334198

80152175407

1384838224

6214278

6214278

460147

6006

400141

1271644

227029

227029

701926

701926

5718093227701550381049617

365505267827912117140

365505267827912117140

365505267827912117140

528221266826122999918046

76821

76821

4064519470

527806266775122947917528

451046

92353175494117351

7884021911381

180614578014064

26201864

514680261511116339892530

1415635

658443381040

486139208833

6811105184

202527

202527

907792591245

0178

0178

83245116993

83245116993

717280

216770

50510

683264164

1820240

501242104

002020

0202

0202

0202

10110102

10110102

10110102

577324406

25696154

0178

25589146

0101

0101

318228249

0101

0101

0101

0101

255930011253984

6634859770

6634859770

189625210663214

189625210663214

1001

1001

1001

11228131

0066

0022

0044

11222125

187025

187025

187025

1382447209

1361130177

0044

78217

620062

361037

2002

1202436

170017

0202

231722

010010

010010

3514052531009

066066

065065

0101

351339253943

351339229919

002424

2957224921117317

267380131778

214933975140342101586

214933975140342101586

0549

0101

0101

0044

0404

0404

214933974640338101577

20621391983964399462

7204231752556955948

556046161

104882140

0101

13913

12327114281017733932

719163224404791

0066

10116384

37143108288

0011

10313273

0123

235260310553893

13413690

0201434

0134

01090109

01090109

0011

0011

8118545941

44199347590

0257

001717

091423

051318

0011

9182249

35165275475

1693459

1693459

1146268415

1137265403

0224

0718

3644147452

16655147818

16655147818

16655147818

0819

0819

1045

1045

1534521381

1431821353

1506

022022

026420284

0101

0112

0123

026117278

0202

03687123

03687123

001414

001414

273224109586100

2475012309

2475012309

0011

0011

0011

222479278

222479278

0606

0099

041041

22200222

212225

4105

4105

4105

511105166

243422509415625

243422509415625

243422509415625

234722457785370

001717

390039

0033

150015

1001

1056

223722446455126

54183138

002525

2944477

0213

2914373

0101

580260

580058

0022

01117118

29967159525

063063

0101

062062

062062

062062

062062

910091

910091

910091

910091

880088

3003

00111111

00111111

00111111

00111111

12107128

87440131

034043

034043

034043

003737

0303

0033

871088

0011

0011

0011

0011

0011

39214571994577

39214571994577

002727

002727

0077

0077

002020

001414

0066

39214571724550

10201103

10201103

10200102

0011

38194571714447

38194571714447

38194571584434

001010

0033

678185233

0101

011011

011011

011011

011011

011011

6214884

6214884

6214884

013316

013215

0011

480149

141419

141419

0101

2507

0101

0101

0044

3127388

3127388

3127388

3127388

1001

004444

0022

021315

0044

0011

0101

2103

06915

0202

0606

0101

028028
